# Fetal akinesia deformation sequence with pontocerebellar hypoplasia, and migration and gyration defects

**DOI:** 10.4322/acr.2021.323

**Published:** 2021-09-03

**Authors:** Meghan Elizabeth Kapp, Pamela Lyle, Hilary Highfield Nickols

**Affiliations:** 1 Vanderbilt University Medical Center, Department of Pathology, Microbiology and Immunology, Nashville, TN, USA; 2 C.W. Bill Young VA Medical Center, Department of Pathology & Laboratory Medicine Services, Bay Pine, FL, USA; 3 Norton Healthcare, CPA Laboratory, Louisville, KY, USA

**Keywords:** arthrogryposis, corpus callosum, infant, newborn, karyotype, phenotype

## Abstract

Fetal akinesia deformation sequence (FADS), or Pena-Shokeir phenotype is a constellation of deformational changes resulting from decreased or absent fetal movement, and include arthrogryposis, and craniofacial and central nervous system anomalies. We report an autopsy case of a 36-6/7week female neonate with a normal female karyotype and chromosome microarray demonstrating findings consistent with FADS. We provide a detailed examination of the severe and complex central nervous system abnormalities, including marked pontocerebellar hypoplasia and cortical and cerebellar migration and gyration defects. This case represents a rare detailed examination of the central nervous system of a patient with FADS.

## INTRODUCTION

Fetal akinesia deformation sequence (FADS) or Pena-Shokeir phenotype (PSP) is a lethal condition first described in 1974 by Pena and Shokeir.^1^ The defining feature of this heterogeneous disorder is deformational abnormalities due to decreased or absent fetal movement, e.g., arthrogryposis, fetal growth retardation, polyhydramnios, and pulmonary hypoplasia.^2^^-^4 The incidence of PSP is estimated to be 1:12,000 births, although a high number of cases may go unreported.^5^ The terms Pena-Shokeir phenotype and FADS are not diagnoses, but rather a synopsis of multiple symptoms and clinical features that require further investigation into the underlying cause. Detailed descriptions of the neuropathology, specific positioning, and somatic anomalies has since allowed for the subcategorization of distinct entities in sporadic and familial cases.^6^ Additionally, genetic mutations associated with specific variants of FADS have been identified, fostering the study of fetal movement and pathogenetic mechanisms for the phenotypic findings. We therefore present, with IRB approval (VUMC IRB 201141), an in-depth study of a rare case of a newborn with FADS with marked central nervous system (CNS) abnormalities, including pontocerebellar hypoplasia and cortical and cerebellar migration and gyration defects in the setting of normal genetic microarray.

## CASE REPORT

The patient was a female neonate, born at 36 6/7 gestational weeks, via scheduled Cesarean section for fetal anomalies and malposition, including neck hyperextension, detected on ultrasound. The maternal history was remarkable for gestational diabetes, and involvement in a motor vehicle accident at 12 gestational weeks. Beginning at 18 5/7 gestational weeks, the prenatal ultrasounds demonstrated multiple fetal anomalies including absent corpus callosum, severe ventriculomegaly, arthrogryposis with bilateral talipes, and kyphoscoliosis. At delivery, the umbilical cord was wrapped once around the neck and twice around the chest. The infant expired shortly after birth due to respiratory failure.

## AUTOPSY PRESENTATION

**Figure 1:**
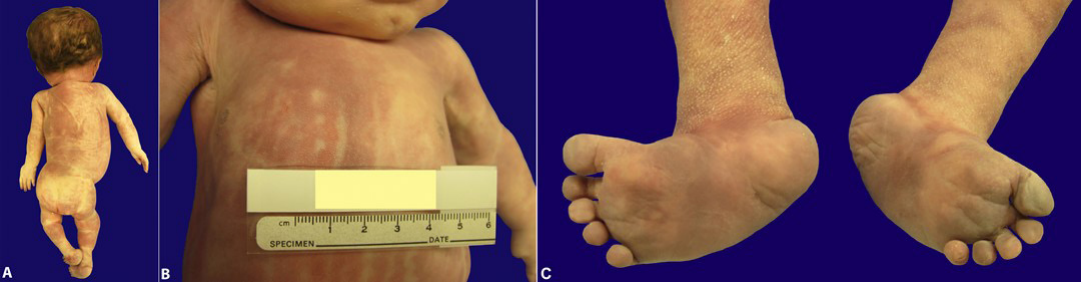
Gross photographs of somatic findings show **A** – kyphoscoliosis, macrocephaly, short neck, and sacral dimple; **B** – laterally displaced right nipple; and **C** – rocker-bottomed feet.

The postmortem examination confirmed the prenatal imaging findings of multiple and complex somatic and CNS anomalies. The external somatic findings include kyphoscoliosis, short neck, and macrocephaly (head circumference 37.5 cm; expected for age, 32.5 +/- 1.6 cm) (Figure 1A) with facial dysmorphia consisting of a shortened, blunt nasal bridge and flattened nose, hypertelorism, and low set and posteriorly rotated ears, and right ear accessory tragus. The chest demonstrated a laterally displaced right nipple (Figure 1B) and the back had a sacral dimple (Figure 1A). The limbs demonstrated arthrogryposis (Figure 1A), multiple lower limb fractures, visualized on postmortem radiographs without reported history of difficult delivery, rocker-bottomed feet (Figure 1C), and adducted thumbs.

Internal examination revealed fused ribs, cardiomegaly (weight 26 grams, expected 16.7 +/- 5.4 grams), enlarged thymus (weight 23.2 grams, expected 8.4 +/- 5.6 grams), and polysplenia. The placenta was small for gestational age (weight 320 grams, expected for age, 467 +/- 107 grams) with increased fetal:placental weight ratio of 9.2 (expected 6.8 +/- 1.1). Placental disc demonstrated focal necrosis at the site of circumvallate membrane insertion, and patchy villous edema and uneven villous maturation. Fetal membranes with circumvallate insertion but no significant histopathologic changes. The umbilical cord showed three vessels with no significant histopathologic changes. Conventional cytogenetics showed a normal female karyotype. A chromosome microarray (V6 Oligo) was also normal. This microarray detects all cytogenetically defined microdeletion and microduplication syndromes, copy number changes greater than 100 kb, significant exonic changes of selected genes in the nuclear genome, and deletions greater than 2 kb in the mitochondrial genome. It does not detect balanced translocations, inversions, low level mosaicism, point mutations, uniparental disomy, imprinting defects or genomic imbalances in regions that are not represented.

**Figure 2:**
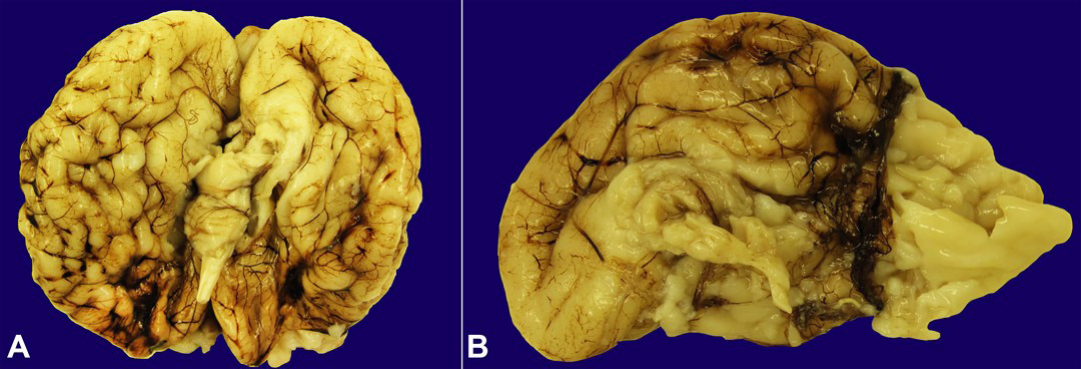
Gross photographs of the brain demonstrate severe and complex malformations including generalized pachygyria of the cerebral cortex bilaterally. **A** – ventral surface; **B** – medial right frontal lobe and total agenesis of the corpus callosum.

**Figure 3:**
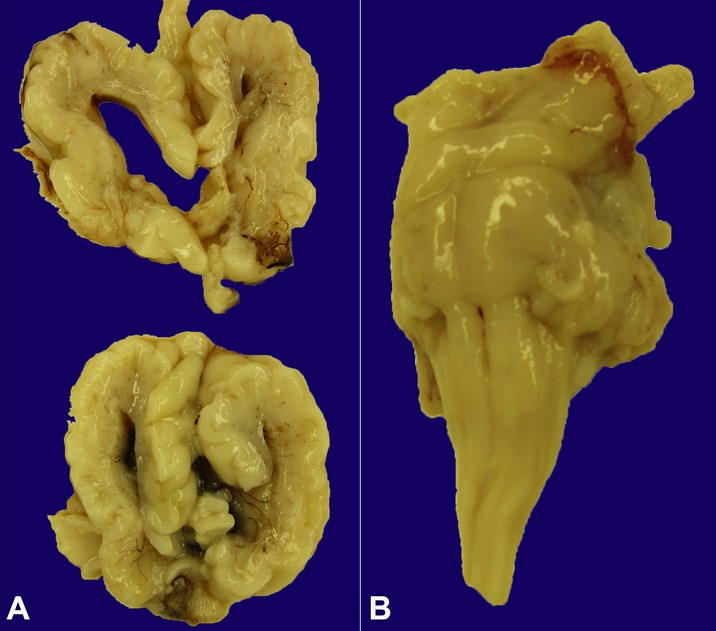
Gross photographs of the brain demonstrate marked ventriculomegaly: **A** – coronal sections with lateral enlarged lateral ventricles. The brainstem was hypoplastic: **B** – posterolateral, including the midbrain and pons.

Gross neuropathologic examination demonstrated a 323 g brain (appropriate for gestational age: 298 ± 70 grams) with generalized pachygyria (Figure 2A, B) of the cerebral cortex bilaterally, total agenesis of the corpus callosum (Figure 2B), and marked ventriculomegaly (Figure 3A). The brainstem was hypoplastic overall (Figure 3B). The midbrain and pons were both hypoplastic and the cerebellum was hypoplastic overall with the vermis and lateral hemispheres present; no cerebellar cysts were identified. The hippocampus was not identified, suggesting agenesis.

**Figure 4:**
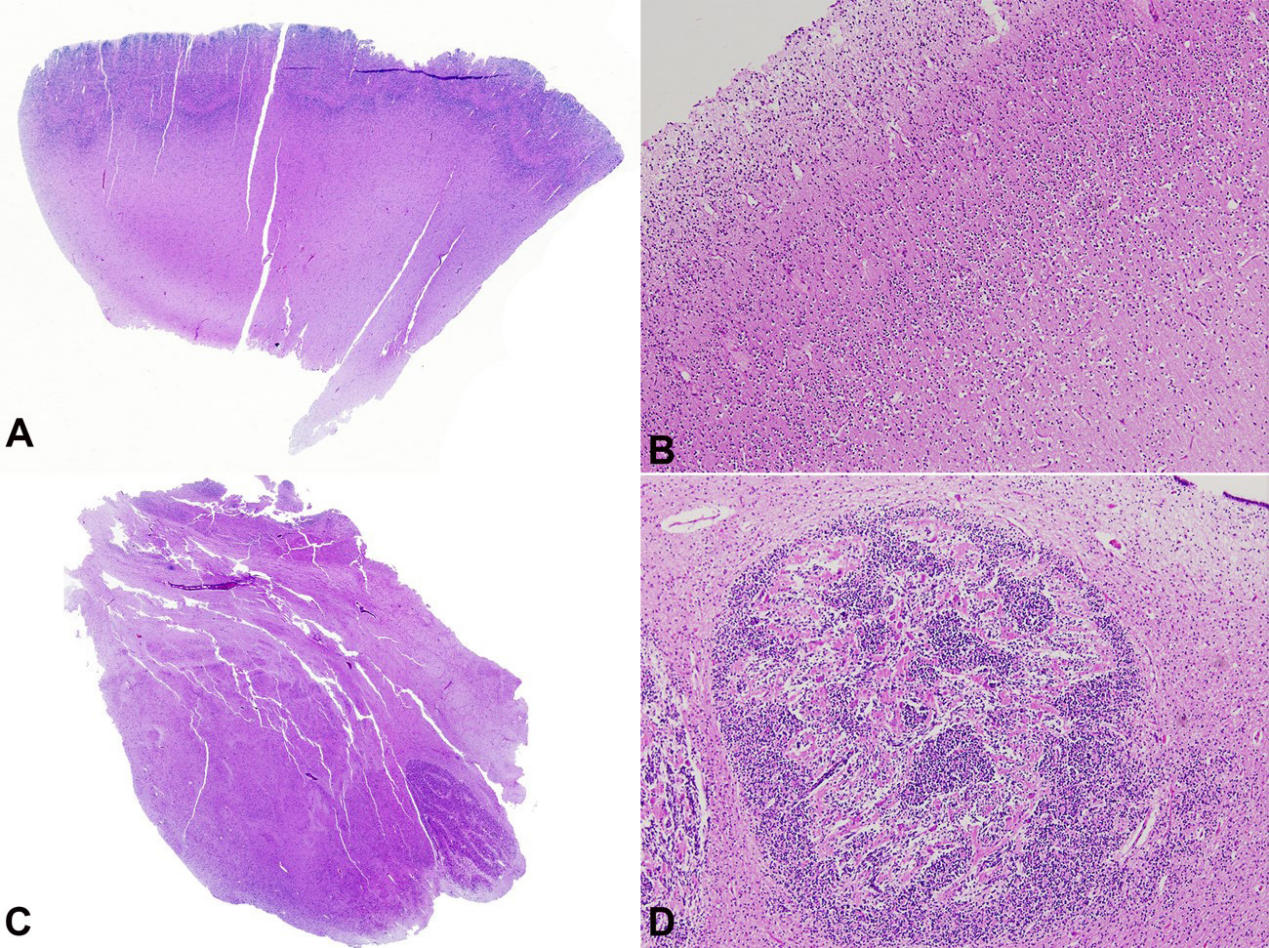
Microscopic neuropathologic examination demonstrated a smooth cerebral cortical surface due to lack of sulcation, consistent with pachygyria (**A** –1X). Within the molecular layer (Layer I), there were nodular heterotopia comprised of small neuroblastic-looking cells and complete disorganization of the underlying cortex, with no distinct lamination (**B** – 40X). The lateral geniculate nucleus in the thalamus was hypertrophic (**C** – 1X)**.** Heterotopia comprised of mature neurons were present in the cerebellar white matter (**D** – 200X).

Microscopic neuropathologic examination demonstrated a smooth cerebral cortical surface with lack of sulcation, consistent with pachygyria (Figure 4A) with large glioneuronal heterotopia in the overlying leptomeninges. There was no subarachnoid hemorrhage, inflammation, or cyst formation. Within the molecular layer (Layer I), there were nodular heterotopia comprised of small neuroblastic-looking cells (Figure 4B). The underlying cortex was completely disorganized, with no distinct lamination. The neurons were inappropriately immature, without apparent differentiation into granule cells or pyramidal cells. There was no neuronal loss, gliosis, or inflammatory infiltrates, and neuronal storage material was not identified. Diffuse gliosis of mild-to-moderate severity was present in the cerebral white matter. A single focus of chronic/remote periventricular leukomalacia (PVL) was identified upon histologic examination in the deep white matter; it was comprised of a healing necrotic focus with mineralized axons, gliosis, and macrophages surrounded by diffuse gliosis in the white matter. Periventricular nodular heterotopia, comprised of misplaced neurons, were identified. The lateral geniculate nucleus in the thalamus was hypertrophic (Figure 4C). The interstitial white matter neurons did not appear increased. There were no white matter hemorrhages. The germinal matrix lining the lateral ventricles was neither hemorrhagic nor gliotic.

The cerebellar cortex was markedly malformed. The extreme lateral hemispheric folia were not formed. While the medial part of the lateral hemisphere contains folia, they appear small in size. In these folia, the external granular layer was approximately 8 cells thick (age-appropriate), and the Purkinje cells and internal granular cells were present in proper laminated formation. There is no obvious acquired loss of Purkinje cells or Bergmann gliosis in these regions. However, in several regions of the medial hemispheric cortex, certain folia appear dysplastic with disorganized laminae. The vermis was malformed, comprised of a mass of neurons. Heterotopia, comprised of mature neurons, were present in the cerebellar white matter (Figure 4D). There are no cysts, hemorrhages, infarcts, or inflammatory infiltrates. The dentate nucleus was hyperconvoluted; its medial aspect and the medial roof nuclei formed clumps of neurons. In the white matter of the hypoplastic cerebellar hemisphere, there was a medium-sized arteriole with a recanalized thrombus.

The rostral midbrain (level of oculomotor nucleus), as well as caudal midbrain at transition with rostral pons (isthmus) were examined. The aqueduct of Sylvius was patent. The oculomotor nuclei, substantia nigra, dorsal raphe, superior colliculus, and mesencephalic reticular formation were identified and appeared in their proper anatomic positions. There was no gliosis and/or apparent neuronal loss. Hypoplasia of the ventral pons (basis pontis) was striking, especially in comparison to an age-matched control. There is focal gliosis unilaterally associated with pericellular vacuolation. There is a small heterotopia within the middle cerebellar peduncle and in the region of the lateral tegmental nucleus. The ventral cochlear nucleus was displaced dorsally and rostrally, abnormally positioned in the rostral pons. The pararaphales was distinctly prominent. The locus ceruleus, trigeminal nucleus, facial nucleus, pontis oralis and caudalis, and median raphe were identified and appropriately positioned. The ventral lamellae of the principle inferior olive of the medulla oblongata were hypoconvoluted and abnormally thick compared to the dorsal lamella. There was mild neuronal loss and gliosis in the inferior olives. The hypoglossal nucleus, nucleus of the solitary tract, dorsal motor nucleus of the vagus, nucleus gracile, nucleus cuneatus medialis, and reticular formation were identified and appeared appropriately positioned. The thoracic spinal cord was microscopically intact. There was no loss or underpopulation of anterior horn motor neurons in the spinal cord. Skeletal muscle represented by neck strap muscles, lower extremity muscle, and intercostal muscle showed no significant histopathologic change.

## DISCUSSION

The neuropathology of this late preterm female newborn with FADS who died within hours of delivery is complex with combined malformative and acquired vascular-related abnormalities, evidenced by pachygyration and dysplasia of the cerebral cortex as well as the cerebello-ponto-olivary circuitry, and chronic/remote periventricular leukomalacia (PVL) in the cerebral white matter, respectively.

A distinguishing feature in this case is pontocerebellar hypoplasia. Pontocerebellar hypoplasia (PCH) as a diagnostic category encompasses a clinically and genetically heterogeneous group of seven disorders (PCH 1-7).^7^^-^10 As in our case, the disorders begin prenatally, especially in PCH1, PCH2, PCH4, and PCH5. In some milder cases, cerebellar images suggest a perinatal or early postnatal onset.^8^ Of note, PCH1 is associated with anterior horn cell loss and PCH2 and PCH7 with “progressive atrophy” of the cerebral cortex, neither of which are features of our case. Additionally, none of the PCH subtypes are associated with a cortical malformative lesion, as in our case. Interestingly, the genes associated with the PCH subtypes are involved in protein synthesis, particularly in the shared function of tRNA processing, such that a defect in protein synthesis seems one of the most likely biologic mechanisms involved in PCH.^8^ In short, there does not appear to be a complete match of the findings in our patient with those cases cited.^8^^,^^11^

The presence of central nervous system dysgenesis in the setting of normal tissue microarray and cytogenetics argues against a synaptopathy as the cause of the FADS in this case. Neuromuscular junction disease results from disruption of the signal transduction by presynaptic acetylcholine transferase, acetylcholine esterase in the synapse, and the postsynaptic muscular nicotinic acetylcholine receptor. The functional acetylcholine receptor is comprised of multiple proteins including muscle skeletal tyrosine kinase (MUSK), the muscle-intrinsic activator of MUSK (DOK7), agrin, and rapsyn. Mutations of the acetylcholine receptor pathway have been reported to result in FADS^12^ including a Dutch founder mutation in MUSK reported by Tan-Sindhunata et al,^13^ and a germline mutation in DOK7 by Vogt et al.^14^ A specific gene associated with our case of FADS with pontocerebellar hypoplasia is not readily apparent. In terms of a genetic disorder, a family pedigree in our patient is not available to us, but is critical towards defining a genetic role in this particular case. Key information in this regard is, however, the normal female karyotype and chromosome microarray.

In our patient, there is histopathologic evidence of remote hypoxic-ischemic injury, with healed mineralized PVL in the cerebral white matter, and a recanalized thromboembolus in a medium sized arteriole in the cerebellar white matter. Because the infant died shortly after birth, these lesions necessarily occurred *in utero.* These two findings raise the possibility of primary hypoxic-ischemic injury early in gestation that may have contributed to the malformative processes in the cerebral cortex, cerebellum, and brainstem.^15^^-^17 This formulation, however, is speculative. Central nervous system dysgenesis in a background of hypoxia-induced injury encompassing mineralized neurons and microinfarcts with or without loss of anterior horn neurons, and polymicrogyria are frequently reported within the literature. Rudzinski et al^18^ note that these CNS findings are often reported in the absence of histologic descriptions of skeletal muscle findings. In their case series, Rudzinski et al^18^ note CNS findings suggestive of hypoxic/ischemic injury were present in 4 of 6 (66%) evaluable fetuses with arrested skeletal muscle maturation.^18^ The latter, described as myotubular morphology characterized by linear chains of central nuclei, is often incorrectly interpreted as a primary myopathy when actually resulting secondarily to CNS hypoxia. Thus, they suggest a myopathic etiology ought to only be ascribed in the absence of CNS pathology. The strap muscles of the neck and skeletal muscle of the leg in our case demonstrate a normal histomorphology.

## CONCLUSION

Our case, including data of the pregnancy history, did not disclose general etiological factors. The role of maternal gestational diabetes associated with FADS,^11^ and/or car accident with possible uterine/fetal trauma in the pathogenesis of the FADS in our patient is completely unknown. We report this case in order to continue to define the heterogeneity of the FADS and the different genetic and/or acquired disorders that cause it in order to better refine our understanding and clinical care of affected families.
